# External validation of the REMEMBER score

**DOI:** 10.3389/fcvm.2023.1192300

**Published:** 2023-07-28

**Authors:** Armin Darius Peivandi, Henryk Welp, Sebastian Kintrup, Nana Maria Wagner, Angelo Maria Dell’Aquila

**Affiliations:** ^1^ Department of Cardiothoracic Surgery, University Hospital Muenster, Muenster, Germany; ^2^ Department of Anesthesiology, Intensive Care and Pain Therapy, University Hospital Muenster, Muenster, Germany

**Keywords:** extracorporeal life support, coronary artery bypass grafting, score system, cardiac failure, risk score

## Abstract

**Background:**

The use of venoarterial extracorporeal membrane oxygenation (VA-ECMO) after coronary artery bypass grafting (CABG) is associated with high in-hospital mortality rates. The pRedicting mortality in patients undergoing venoarterial Extracorporeal MEMBrane oxygenation after coronary artEry bypass gRafting (REMEMBER) score has been created to predict in-hospital mortality in this subgroup of patients. The aim of this study is to externally validate the REMEMBER score.

**Methods:**

All CABG patients who received VA-ECMO during or after the operation at our center between 01/2012 and 12/2021 were included in the analysis. Discrimination was assessed using concordance statistics, visualized by ROC curve analysis. Calibration-in-the-large and Calibration slope were tested separately.

**Results:**

A total of 107 patients (male: *n* = 78, 72.9%) were included in this study. The in-hospital mortality rate in our cohort was 45.8% compared with 55% in the original study. The REMEMBER score median predicted mortality rate was 52% (76.9–36%). However, the REMEMBER score showed low discriminative ability [AUC: 0.623 (*p* = 0.0244; 95% CI = 0.524–0.715)] and inaccurate calibration (intercept = 0.25074; *p* = 0.0195; slope = 0.39504; *p* = 0.0303), indicating poor performance.

**Conclusions:**

The REMEMBER score did not predict in-hospital mortality and was therefore not applicable in our cohort of patients. Additional external validation studies in a multicenter setting are therefore advisable.

## Introduction

### Background and objectives

Postcardiotomy extracorporeal life support is associated with high in-hospital mortality rates. In a retrospective analysis, Raffa et al. found that the survival-to-discharge rate in patients was only 37% (1). Their retrospective study included not only patients who had undergone coronary artery bypass grafting (CABG) but also those who had undergone valve and aortic surgeries. The in-hospital mortality rate for the subgroup of patients receiving venoarterial extracorporeal membrane oxygenation (VA-ECMO) after CABG was equally high. In a meta-analysis, including an analysis of the data from 12 centers, the in-hospital mortality rate was 64.2% (2).

Furthermore, VA-ECMO therapy is associated with huge costs for the healthcare system. Recently, it was shown that the median cost of a hospital stay with ECMO therapy exceeded 100,000 $ per patient (3).

In light of these issues, a scoring system to identify patients who are likely to profit from VA-ECMO after CABG could be a beneficial tool for clinicians.

In order to predict in-hospital mortality in patients undergoing VA-ECMO after CABG, the pRedicting mortality in patients undergoing venoarterial Extracorporeal MEMBrane oxygenation after coronary artEry bypass gRafting score (REMEMBER score) was introduced recently. This score has six pre-ECMO parameters: older age, left main disease, inotropic score > 75, CK-MB > 130 IU/L, serum creatinine > 150 µmol/L, and platelet count < 100 × 10^9^/L. In the REMEMBER score mentioned above, score calculation was based on 166 patients from a single center, and the results showed good discrimination (AUC 0.85, 95% CI 0.79–0.91) and calibration (Hosmer–Lemeshow *χ*^2^ *p* = 0.644) in this cohort (4).

Hence, the REMEMBER score is designed as an assessment instrument to select patients who can potentially benefit from VA-ECMO after CABG.

However, construction data of the REMEMBER score were obtained from only one institution. Therefore, a broader and potential worldwide application remains to be assessed.

In this regard, we aim to externally validate the REMEMBER score in our cohort of patients who received VA-ECMO after CABG.

## Material and methods

### Source of data/study design

This study is a retrospective, external validation study of a previously published scoring system (4). TRIPOD criteria provided the basis for this study and article structuring (5).

### Participants

The reported validation is a single-center analysis. All CABG patients who received VA-ECMO during or after the operation at our center between 01/2012 and 12/2021 were included in the analysis.

### Outcome

The outcome, predicted by the REMEMBER score, was in-hospital mortality.

### Predictors

Score variables were age, left main disease, inotropic score > 75, CK-MB > 130U/L, serum creatinine > 150 µmol/L, and platelet count < 100 × 10^9^/L. Points were classified as weighted and assigned in the original score publication (4). The lowest values recorded within 6 h before ECMO implantation were considered for analysis. If the laboratory values were not measured within the last 6 h before ECMO (elective patients), the last values before implantation were taken for score calculation.

### Missing data

Patients with missing data (missing variables for score calculation) (*n* = 11) were excluded from the analysis.

### Sample size

Exclusion criteria were adapted in accordance with the original score publication (age <18 years, VV-ECMO, ECMO before CABG operation or more than 7 days after operation, and concomitant major cardiac surgeries) (4). After the exclusion process, our study included a total of 107 patients.

### Statistical analysis methods

Patient characteristics were reported as either *n* (%), median (Q 3–1), or mean (std). The normality of continuous variables was assessed using the Shapiro–Wilk test. The patients were divided into four risk groups (I: lowest risk, IV: highest risk) as described in the original study (4). In-hospital mortality rates of the different risk groups were assessed. The ABCD model for validation was used for score analysis (6). For calibration analysis, the model proposed by Cox et al. (7) was used. Calibration-in-the-large and Calibration slope were assessed. In this regard, the predicted mortality rate was obtained using the prediction formula described in the original score analysis [1/(1 + exp. (3.856–0.220 * score)] (4). Discriminative score ability was quantified using concordance (C)—statistic, visualized by a ROC curve.

### Ethical approval

The local ethics committee (“Ethikkommission der Ärztekammer Westfalen Lippe und der Westfälischen Wilhelms Universität”) provided ethical approval for a retrospective analysis of ECMO patients (2015-547-f-S). Because of the retrospective nature of the study design, informed consent of each individual was not needed and was therefore waived.

## Results

### Participants

A total of 107 patients (male: *n* = 78, 72.9%) with a median age of 68 (Q 3–1: 74–62) who received VA-ECMO during or after CABG surgery between 2012 and 2021 were included in our study. Unfortunately, 49 patients (45.8%) died during their hospital stay. Those who underwent left coronary artery main stenosis accounted for 54.2% (*n* = 58). In 55 patients (51.4%), VA-ECMO was implanted intra-operatively, and in 52 patients (48.6%), implantation was done after surgery. Detailed patient characteristics and score variables are summarized in Table 1.

**Table 1:** Patient characteristics pre-VA-ECMO (score constructing variables: lowest values 6 h pre-ECMO) and in-hospital mortality.

Characteristic	Median (Q 3–1), mean (std), or *n* (%)
Age	68 (74–62)
Male sex	78 (72.9)
CK-MB (IU/L)	65 (196–22.5)
Serum creatinine (µmol/L)	97.2 (159.1–79.6)
Platelet count (×10^9^/L)	200 (263.5–149)
Inotropic score	50 (83–30)
Duration of ventilation (h)	126 (358–58)
ICU stay (day)	18 (37–8)
Duration of operation (min)	261 (336.5–205)
ECC duration during operation (min)	125 (155.75–100.25)
X-clamp time (min)	64.5 (79–50.25)
Emergency surgery	67 (62.6)
Number of distal anastomoses	3 (4–3)
Cannulation of VA-ECMO	
• Central	67 (62.6%)
• Peripheral	40 (37.4%)
EuroSCORE	8 (11–5)
LogEuroSCORE	9.73 (20.89–4.48)
CPR	22 (20.6)
In-hospital mortality	49 (45.8)
REMEMBER Score variables	
REMEMBER Score	18.3 (6.7)
Age < 54 years	7 (6.5)
Age 54–67 years	46 (42.9)
Age > 67 years	54 (50.5)
Left main disease	58 (54.2)
Inotropic score >75	27 (25.2)
CK-MB > 130 IU/L	37 (34.6)
Serum creatinine > 150 µmol/L	30 (28)
Platelet count < 100 × 10^9^/L	10 (9.3)

### Model performance

**Figure 1:**
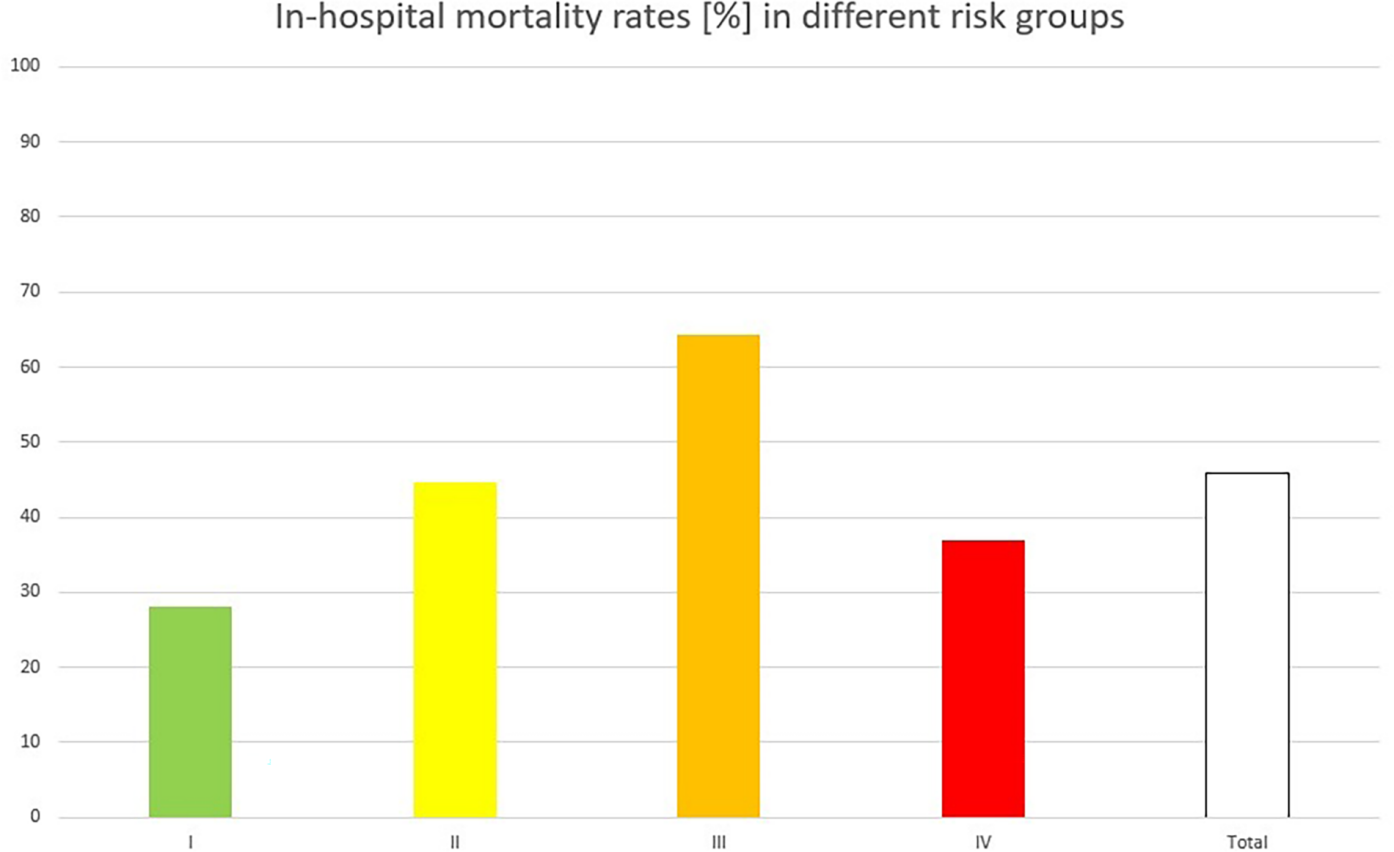
Observed in-hospital mortality rates in different risk groups, built according to the original study from I (lowest risk) to IV (highest risk): risk group IV showed a lower in-hospital mortality rate than risk groups II and III, indicating a mortality overestimation by the score in higher values.

The REMEMBER score median predicted mortality rate in our cohort was 52.6%. The observed mortality rates in the risk groups were I: 28%, II: 44.7%, III: 64.3%, and IV: 36.8%. This means that in-hospital mortality in the highest risk group (IV) was lower compared with risk groups II and III (Figure 1). This indicates overestimation of the scores in high values.

**Figure 2:**
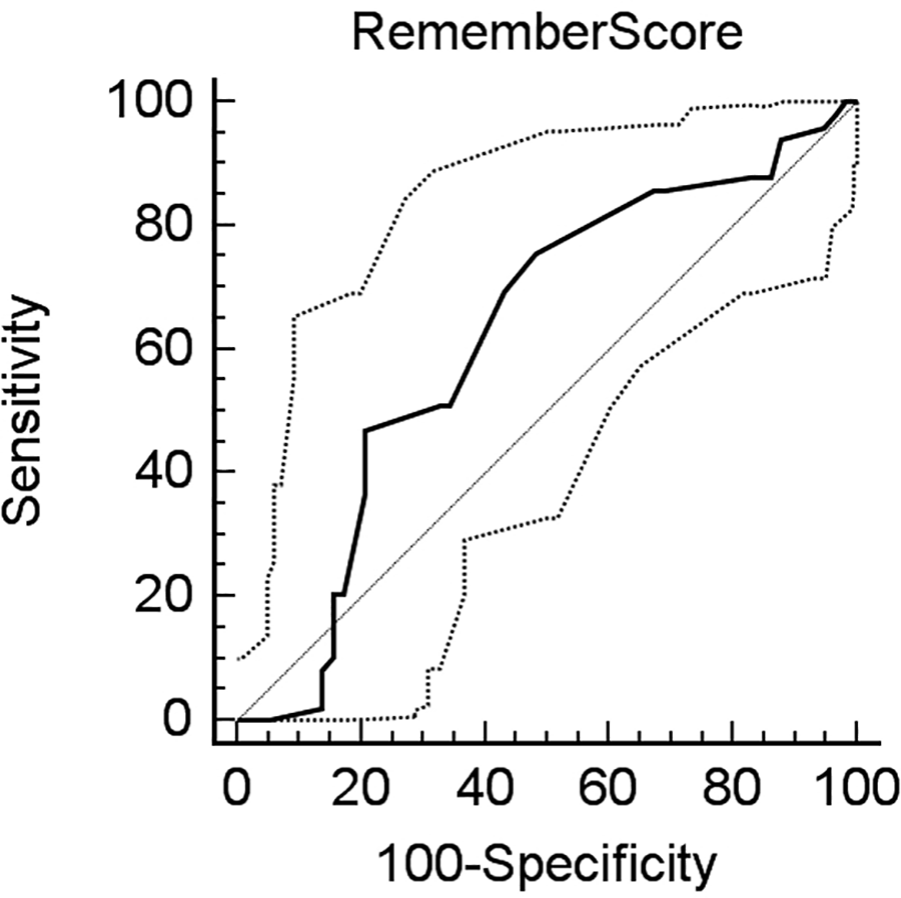
ROC curve showing low discrimination of the REMEMBER score in our cohort [AUC of 0.623 (*p* = 0.0244; 95% CI = 0.524–0.715)].

Figure 2 shows the REMEMBER score discrimination in our cohort of patients. Although the score median predicted mortality was close to observed mortality, the score showed a low discriminative ability with an AUC of 0.623 (*p* = 0.0244; 95% CI = 0.524–0.715.

**Figure 3:**
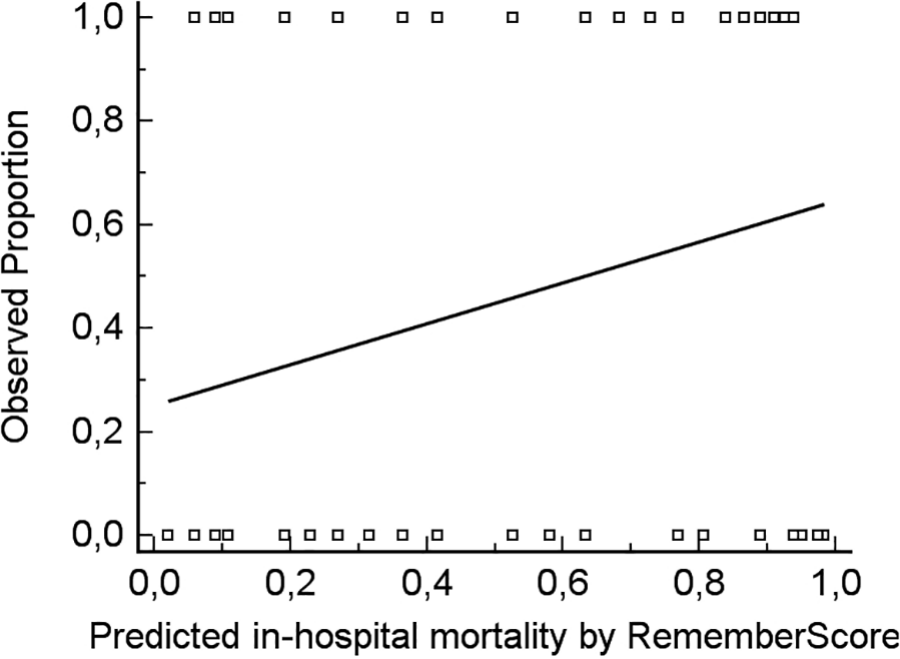
Calibration curve, demonstrating that low prediction values of the REMEMBER score underestimate mortality, while higher values overestimate the same [calibration-in large (intercept) 0.25074 (std. error 0.10566, *t*-value 2.3731, *p* = 0.0195), Slope 0.39504 (std. error 0.17991, *t*-value 2.1958, *p* = 0.0303)].

As shown in Figure 3, the REMEMBER score was also not accurately calibrated in our cohort. Calibration-in-the-large (intercept) was 0.25074 (std. error 0.10566, *t*-value 2.3731, *p* = 0.0195) and the Calibration slope was 0.39504 (std. error 0.17991, *t*-value 2.1958, *p* = 0.0303). This indicates that low prediction values of the score underestimate mortality, while higher values overestimate mortality.

## Comment

The REMEMBER score was developed to predict in-hospital mortality after VA-ECMO implantation following CABG surgery. Within our cohort of 107 CABG patients who suffered from peri- or postoperative cardiogenic shock requiring VA-ECMO implantation, the score was not able to predict in-hospital mortality.

When discussing the reasons for the poor performance of this score in our cohort of patients, three main issues should be considered:

First, the establishment of a scoring system consists of three essential steps: derivation, validation, and impact analysis (8). While derivation depends on the original population of the score, validation is an ongoing task, comprising external analyses in different study populations. In our study, we intended to provide the basis for such a validation process. As no population of patients is exactly equal, we also noted some differences in the baseline characteristics of our patients.

In comparison with the development data of the REMEMBER score, the patients in our cohort were older (68 vs. 61 years). They had a higher incidence of left main disease (54.2% vs. 31%), a lower median CK-MB (65 vs. 143 IU/L), a lower median serum creatinine level (97.2 vs. 121 µmol/L), almost equal median platelet counts (200 vs. 203 × 10^9^/L), and a lower median inotropic score (50 vs. 75). In our cohort, emergency surgery was more common (62.6% vs. 15%). In addition, the collective of Wang and colleagues’ consisted of 83 (50%) off-pump CABG patients, whereas almost every patient in our collective had undergone cardiopulmonary bypass [n_OPCAB_ = 3, (2.8%)]. In addition, our cohort showed a higher median EuroSCORE (8 vs. 6) and a higher percentage of cardiopulmonary resuscitation (CPR) (20.6 vs. 17%) (4).

The differences, especially in EuroSCORE, on-pump vs. off-pump ratio, and CPR, indicate that our cohort of patients reflects a more typical present-day postcardiotomy VA-ECMO cohort than the original study population. An ideal scoring system should be applicable in such a cohort so that it may become clinically relevant in everyday patient care.

In the REMEMBER score–deriving cohort, VA-ECMO was established through peripheral cannulation. As most of the VA-ECMOs in our cohort were inserted through central arterial cannulation [67 (62.6%)], we performed a subanalysis of peripheral cannulated patients to investigate whether the REMEMBER score was too specific. In this subgroup, however, score discrimination was even worse (AUC: 0.465, 95% CI: 0.306–0.629, *p*: 0.7034).

Although differences in study populations have an impact on score performance, an ideal scoring system should be applicable in all cohorts of plausibly related patients. Thus, external validation is a *sine qua non* for clinical application and, hence, impact analysis. In this regard, the REMEMBER score did not achieve promising results in our cohort of patients.

Second, a question arises as to whether the right variables were chosen for score construction in the first place. Another score that aims to assess survival prognosis in VA-ECMO patients is the PREDICT VA-ECMO score, developed by Wengenmayer and colleagues. This score mainly focuses on pH, lactate levels, and serum bicarbonate concentrations. These three parameters were missing in the REMEMBER score (9). The importance of serum lactate levels as a predictive variable in mechanical circulatory support is also highlighted in a multicenter prospective cohort study by Scolari et al. They found serum lactate and lactate clearance at 24 h to be independent predictors of short-term survival (10). In comparison with the REMEMBER score, both studies focus on predictions made shortly after VA-ECMO implantation. The impact of pre-ECMO lactate levels on ECMO survival remains to be assessed in CABG patients.

The SAVE score, yet another published score for mortality prediction in VA-ECMO patients, comprises 12 different parameters. In contrast to the REMEMBER score, this score includes more perioperative parameters, such as, but not limited to, acute organ failure (liver, central nervous system, kidneys) and respiratory and hemodynamic parameters (11).

In addition, when examining the score constructing variables of the REMEMBER score, it is found that the pre-ECMO platelet count was used as a predicting parameter. A recently published study showed that a relative decrease in platelets on day 1 from ECMO initiation was an independent risk factor for mortality (12). In contrast, major complications resulting from ECMO, such as bleeding and heparin-induced thrombocytopenia, affect the platelet count. In this regard, platelet count serves as a surrogate parameter. Against this background, it becomes questionable whether the pre-ECMO platelet count alone is able to predict mortality. More studies are needed in the future to examine this issue.

Third, perioperative treatment protocols can also have an impact on survival on VA-ECMO after coronary surgery. In this regard, weaning strategies and conditions and a switch to a left-ventricular-assist device (LVAD) are only two examples of confounders affecting survival and therefore misleading score results (13).

In the original REMEMBER score study, 80% of the patients had a running intra-aortic balloon pump treatment prior to VA-ECMO implantation. As this setting leads to improved coronary perfusion and a beneficial reduction in afterload, ECMO implantation might have taken place under more “balanced” circumstances (14). This could have ultimately weakened the mortality prediction of the score once again.

## Conclusion

In our external validation study, we found that the REMEMBER score did not adequately forecast in-hospital mortality. This was true for both discrimination and calibration. The potential reasons for the poor score performance in our patient cohort have been discussed in detail.

## Limitations

This validation study is a single-center study with 107 patients. Additional external validation in different cohorts is needed to conclude whether the REMEMBER score should be verified or dismissed.

## Data Availability

The raw data supporting the conclusions of this article will be made available by the authors, without undue reservation.
